# Flammability Characteristics and Mechanical Properties of Casein Based Polymeric Composites

**DOI:** 10.3390/polym12092078

**Published:** 2020-09-12

**Authors:** Hanbin Lee, Nam Kyeun Kim, Daeseung Jung, Debes Bhattacharyya

**Affiliations:** Centre for Advanced Composite Material, Department of Mechanical Engineering, University of Auckland, Auckland 1010, New Zealand; nam.kim@auckland.ac.nz (N.K.K.); da.jung@auckland.ac.nz (D.J.); d.bhattacharyya@auckland.ac.nz (D.B.)

**Keywords:** casein, inorganic phosphate, composite, char formation

## Abstract

Even though casein has an intrinsic potential ability to act as a flame retardant (FR) additive, the research regarding the FR performance of casein filled polymeric composites has not been thoroughly conducted. In the present work, two commercial casein products, such as lactic casein 720 (LAC) and sodium casein 180 (SC), were chosen to investigate their effects on the performances of the polypropylene (PP) composites. The melt compounding and compression moulding processes were employed to fabricate these casein-based composites. Ammonium polyphosphate (APP) was also selected to explore its combined effects in conjunction with casein on the composite’s flammability. The cone calorimeter results showed that the addition of casein significantly reduced (66%) the peak heat release rate (PHRR) of the composite compared to that of neat PP. In particular, the combination of LAC and APP led to the formation of more compact and rigid char compared to that for SC based sample; hence, a further reduction (80%) in PHRR and self-extinguishment under a vertical burn test were accomplished. Moreover, the tensile modulus of the composite improved (23%) by the combined effects of LAC and APP. The overall research outcome has established the potential of casein as a natural protein FR reducing a polymer’s flammability.

## 1. Introduction

The usage of natural materials in the composites field has become vital because of the growing needs for sustainable production, coupled with the increasing environmental awareness [1]. In particular, cellulosic fibres have been seriously investigated as reinforcements to replace the synthetic fibre reinforcement due to their certain merits, such as reproducibility, low pollutant emission, biodegradability and high specific stiffness and strength [2]. However, high flammability characteristics of these fibres have limited the use of their composites where fire protection is required. The current research has been carried out to overcome some of the deficiencies and extend the applicability of the casein-based composites.

Materials based on protein have been investigated in various areas because of their tenability, mechanical properties, biocompatibility, and functionalisation [3]. In particular, the bioengineering field has actively applied the protein-based composites for drug delivery, biosensors, and tissue regenerations. Recently, bio macromolecules including proteins and deoxyribonucleic acid (DNA) have demonstrated their ability to suppress and resist fire under heating [4]. Moreover, the potential of protein-based fibres to reduce flammability of fabrics and polymers have also been explored [2]. Different proteins in fibrous and globular forms, have been used in the substrates, and their ignition and combustion behaviour are determined by the chemical structures and components of the proteins. 

Keratinous fibres (fibrous proteins), such as wool and chicken feather fibres, have been used as reinforcements to improve mechanical and fire-resistant properties of composites. Wool is considered as a flame-retardant (FR) additive due to its relatively high contents of nitrogen and sulphur (3–4 wt%), high ignition temperature (570–600 °C), low heat of combustion (4.9 kcal/g), and high limiting oxygen index (25.2%). Wool fibres in combination with ammonium polyphosphate (APP) have shown a significant reduction in the resulting composite’s flammability with self-extinguishment of fire due to the formation of a rigid and compact char [5,6]. Poly(lactic) acid and polyurethane composites with chicken feather fibres have also demonstrated improved char formation and higher limiting oxygen index [7,8]. Jung and Bhattacharyya [9] have chemically modified wool and feather fibres using a rapid and simple solution treatment process to further improve their fire retardant properties and arrest the mechanical strength drop that is associated with the commonly used fire retardants. The keratinous fibres, chemically treated with intumescent flame retardant (IFR) compounds, have achieved the highest grade of burning resistance, V-0, in a vertical burn test and reduced the peak heat release rate (PHRR) by approximately 33% compared to that of a neat polymer sample.

Unlike the fibrous proteins, globular proteins, such as casein and hydrophobin, have not been investigated in the context of composites. These proteins have mostly been used in the food industry to improve flavour or texture of daily products. In particular, casein-derived peptides have actively been explored to enhance the emulsification properties and solubility in food and health industries [10,11,12]. Recently, casein has also been used as a plastic, synthesised fibre, paint binder, and adhesive in the non-food industries [13,14,15]. Furthermore, casein has been accentuated among researchers due to its flame-retardant ability. Alongi et al. [16] have looked at burning behaviour of casein and DNA and identified the intumescent performance of fire residue under heat radiation. In particular, the carbonaceous residue of casein was significantly expanded during the combustion, but the char formation was unstable with high porosity due to lack of carbon source of casein. Casein and hydrophobin have also been deposited on cotton fabrics to investigate their favourable influences on heat and smoke production of the fabrics [17]. A cone calorimeter experiment has shown that the casein-coated cotton fabric formed the effective char to reduce PHRR by approximately 27% in comparison to the result of the cotton fabric without any treatment. The char formation of casein can be relevant to the presence of phosphoserine clusters in α_s1_/α_s2_ molecules and high content of glutamic acids (~20%) in all casein molecules [18,19]. Even though casein has the effective residue forming ability under heating, the protein has not been employed to investigate its effects on the flammability of polymeric composites. Besides, an effective char formation by the incorporation of casein into polymeric composites can be expected due to carbon supply from polymers during combustion.

To the best of our knowledge, the influences of casein on reducing polymer’s flammability have not been systematically studied. In addition, the research regarding the comparison between different casein products and the combined effects of casein and APP have not been thoroughly conducted. The present paper aims at manufacturing and characterising the casein-based polypropylene (PP) composites to explore the casein’s effects on fire-related and mechanical performances. In particular, two casein products, having different inorganic phosphate contents, such as lactic casein 720 (LAC) and sodium casein 180 (SC), have been selected for a comparative study. The composites have been fabricated using a melt-compounding and compression moulding processes. Fire properties of the composites have been determined by a cone calorimeter and laboratory (UL)-94 burn tests. The composites’ char formation has also been characterised by the optical images of the top and side surfaces. Furthermore, thermogravimetric analysis (TGA) has been carried out to identify the thermal characteristics of the casein and composites under elevated temperatures. The effects of LAC on the mechanical properties of the composites have also been evaluated by conducting tensile tests.

## 2. Experimental Details

### 2.1. Materials

Commercial casein products, lactic casein 720 (LAC) and sodium casein 180 (SC), were supplied by Fonterra (Auckland, New Zealand). The average particle sizes of LAC and SC were 310 µm and 160 µm, respectively. The flame-retardant additive for this research was an ammonium polyphosphate (APP), Budit 3167, having an average particle size of 14 µm (Budenheim, Germany). Polypropylene (PP), HP400N (MFI: 11 g/10 min) from Lyondellbasell, Australia was selected as a polymer matrix.

### 2.2. Composites Fabrication

The composites based on casein and PP were prepared by a compounding and compression moulding processes. Both LAC and SC were dried in an oven at 80 °C for 5 h since the moisture content drops up to around 4% and then dry-blended with PP and APP powder. A melt-blending process of the mixture was carried out using a twin-screw extruder (Brabender GmbH & Co KG, Duisburg, Germany) at the average temperature and a screw speed of 185 °C and 70 rpm, respectively. The compounds were pelletised and then pressed to mould the composite samples (Labtech Engineering, Samut Prakan, Thailand). To prepare the composite samples for fire tests, the pelletised compounds were pressed at the average temperature, pressing load, and pressing time of 185 °C, 5 MPa, and 90 s. For mechanical tests, the average temperature, pressing load and pressing time were set up at 185 °C, 6 MPa, and 150 s. All sheets were cut into the standard sample sizes for the vertical burn, cone calorimeter, and mechanical testing. The compositions of all the samples are described in Table 1. The high content of casein (50 wt%) was selected to identify its sole effects on the thermal and fire-retardant properties of the resultant composites.

### 2.3. Differential Scanning Calorimetery

Calorimetric analyses of all samples have been conducted using differential scanning calorimetry Q1000 (TA instruments, New Castle, DE, US). An empty aluminium pan was used as a reference sample. The differential scanning calorimetry (DSC) test was conducted according to ASTM D3418-15 testing procedures. The samples (~7 mg) were first heated up from 0 °C to 230 °C with a heat rate of 10°C/min and cooled to −20 °C at 10 °C/min. The second heating was then carried out with the same heat rate of the first heating up to 230 °C. The melting temperature (T_m_) of the sample was determined in the second heating and the crystallising temperature (T_c_) of the sample was measured in the cooling. The glass transition temperatures (T_g_) of casein samples, LAC and SC, were evaluated in the second heating.

### 2.4. Thermogravimetric Analysis

Thermal stability and degradation of commercial casein products, a neat PP, and all composites were evaluated by thermogravimetric analysis Q5000 (TA instruments, New Castle, DE, US). A furnace ramp was set up with 10 °C/min and the rate of nitrogen gas was 50 mL/min. All samples were measured from ambient temperature to 700 °C. The weight of samples was set up for APP only at ~7 mg and for other samples at ~20 mg. Temperature at the onset of decomposition (T_onset_) of casein samples were collected at 15% and 5% weight losses for neat PP and all composites. T_max_ was measured at the peak of pyrolysis rate whereas all final residues were analysed at 700 °C.

### 2.5. Flammability Tests

#### 2.5.1. Vertical Burn Test (UL-94)

UL-94 vertical burning test was carried out along with ASTM D3801. Five samples (125 mm × 13 mm × 3 mm) were repeatedly tested in a vertical position to classify different grades, V-0, V-1, V-2, or no rating (NR). The burning behaviour of all samples has been recorded to examine the state of samples during and after tests.

#### 2.5.2. Cone Calorimeter Experiment

Parameters of all composite samples, time to ignition (TTI), heat release rate (HRR), peak heat release rate (PHRR), total heat release (THR), and smoke production, were analysed by Cone calorimeter (Fire Test Technology, East Grinstead, UK) in accordance with ASTM E1354. Three samples (100 mm × 100 mm × 3 mm) were tested in horizontal configuration under the heat flux of 50 kW/m^2^. All the experimental results were used to get the average values of three replicated tests.

### 2.6. Mechanical Tests

A universal testing machine (Instron, Norwood, MA, US) was used to evaluate tensile properties of composite samples with a 5 kN load cell. The test method was followed by ASTM D638 and the testing speed was set up with 5 mm/min. Five samples were prepared as a dog bone shape with approximately 3 mm thickness and 13 mm length, respectively. The chord modulus values were calculated between elongation values of 0.05% and 0.25%.

### 2.7. Environmental Scanning Electron Microscope

The morphology of the composites was characterised by an environmental scanning electron microscope (FEI, Hillsboro, OR, US). Cryofractured surfaces were prepared by liquid nitrogen to identify the dispersion states of additives, such as casein and APP, in the composites. Fractured cross-sections of the composites under tension were observed to investigate the quality of interfacial bonding between the additives and PP.

## 3. Results and Discussion

### 3.1. Differential Scanning Calorimetry

**Figure 1:**
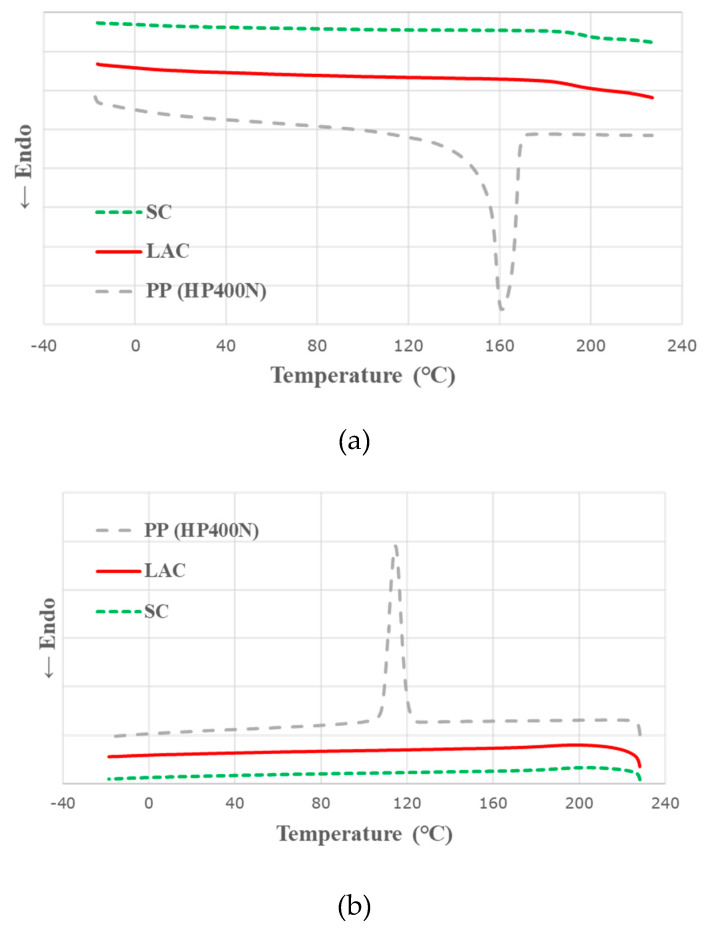
The heating (**a**) and cooling (**b**) curves of neat PP, lactic casein (LAC), and sodium casein (SC) samples.

**Figure 2:**
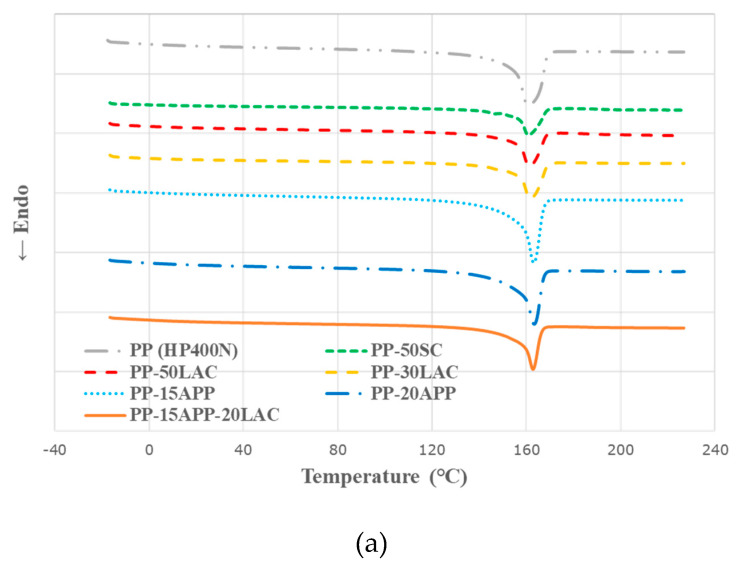

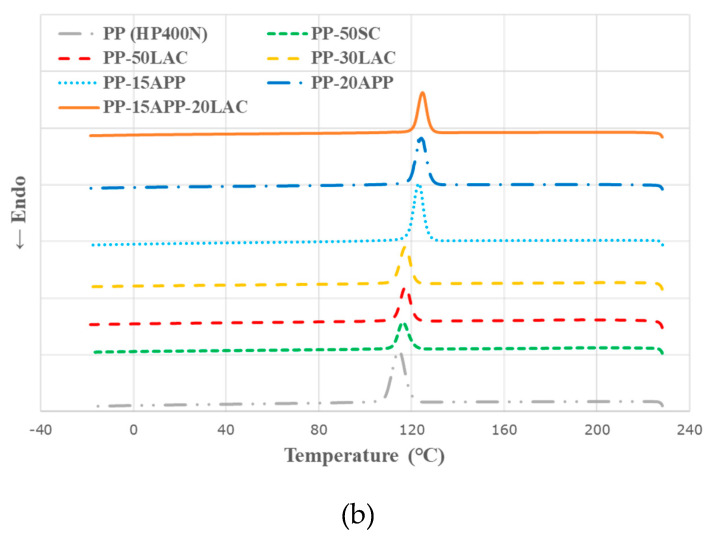
The heating (**a**) and cooling (**b**) curves of neat PP, PP–casein, and PP-ammonium polyphosphate (APP) composite samples.

Differential scanning calorimetry (DSC) curves in Figure 1 show that neat PP has both melting (T_m_) and crystallization (T_c_) temperatures, whereas casein products show glass transition temperatures (T_g_) at ~195 °C. Figure 2 and Table 2 demonstrate that the addition of casein increases both T_m_ and T_c_ of the PP composites compared to those of neat PP. The increase in T_m_ of PP–casein composites can be attributed to the heat stability of the protein (casein) network structure [20]. By contrast, the higher T_c_ values of PP–casein composites can be explained that casein acts as a nucleating agent to facilitate the crystallisation of the composite [21]. The effect of casein on T_c_ can also be identified in PP-15APP-20LAC composite since the incorporation of 20 wt% LAC in PP-15APP promotes an increase of T_c_ of the composite (128.1 °C) in comparison with that of PP-15APP (126.8 °C). On the other hand, the melting point of PP-15APP-20LAC composite indicated that the effect of APP on an increase in T_m_ is more dominant than casein as APP has the long-chain linked by covalent bonds, as opposed to the shell structure of casein micelles.

### 3.2. Thermogravimetric Analysis

**Figure 3:**
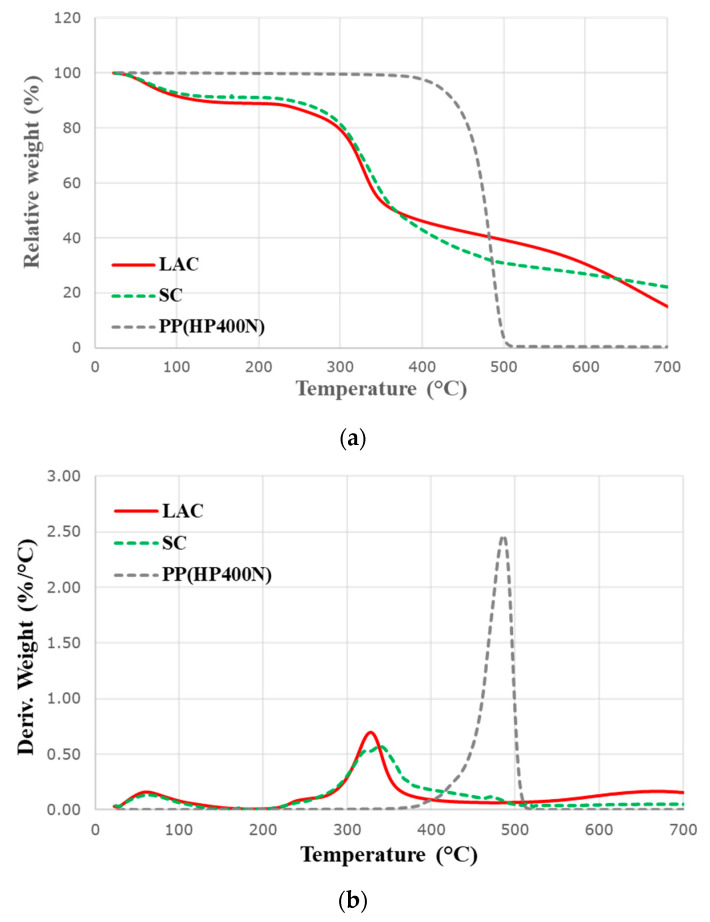
(**a**) Thermogravimetric and (**b**) derivative thermogravimetric curves of neat PP and casein samples in nitrogen.

Thermal degradation of two casein products were analysed by thermogravimetric (TG) and derivative thermogravimetric (dTG) curves in Figure 3a and b, respectively. 

As shown in Figure 3a, LAC and SC have a 10.9% and 8.8% mass loss, respectively at around 100 °C. These losses can be attributed to the moisture evaporation. On the other hand, the TG curve of PP levels off when the main decomposition occurs due to the hydrophobic property of PP. Anca et al. [22] suggested the thermal decomposition of casein in nitrogen as the most possible overall mechanism with two steps at 248–380 °C and 380–680 °C. In the first step, the cleavage of amino acid chains of casein happens in the thermal degradation. Subsequently, the cross-linking and dehydration of the cleaved amino acid chains occurs due to isocyanic acid (HNCO). Simultaneously, carbon monoxide (CO) is evolved. In the second step, carbonaceous residues that are generated in the first step are further decomposed, resulting in producing methane, water, ammonium, and the final char residues. For commercial casein products in this research, it can be said that the major thermal decomposition of both casein commences at approximately 280 °C in Figure 3b and Table 3. Additionally, it can be considered that inorganic phosphate also promotes the cross-linking and dehydration of casein in the first step (248–380 °C) because LAC and SC contain 0.32% and 0.15% of inorganic phosphate, respectively. Figure 3b and Table 3 illustrate that the decomposition rates of both LAC and SC around 335 °C are much lower than that of PP. Besides, it is noted that the decomposition of SC is slower than one of LAC. The effect of sodium on the thermal decomposition of proteins has been reported that the thermal stability of proteins is enhanced due to carboxylate ion complexation [23]. It can be reasonably explained that sodium ionising with carboxyl and sialic acid groups in *C*-terminal blocks of κ-casein molecules in SC delays the decomposition of SC. Both LAC and SC show the increase in the weight percentage of 15.3% and 22.3% carbonaceous residue at the end of tests, respectively compared to that of PP, Figure 3a, and Table 3. The chemical components, such isocyanic acid, inorganic phosphate, and carboxyl/sialic acid groups, in casein react during the thermal decomposition to promote the dehydration and cross-linking processes for the char formation. The slightly lower amount of the LAC can be related to the further decomposition of residue at around 670 °C.

**Figure 4:**
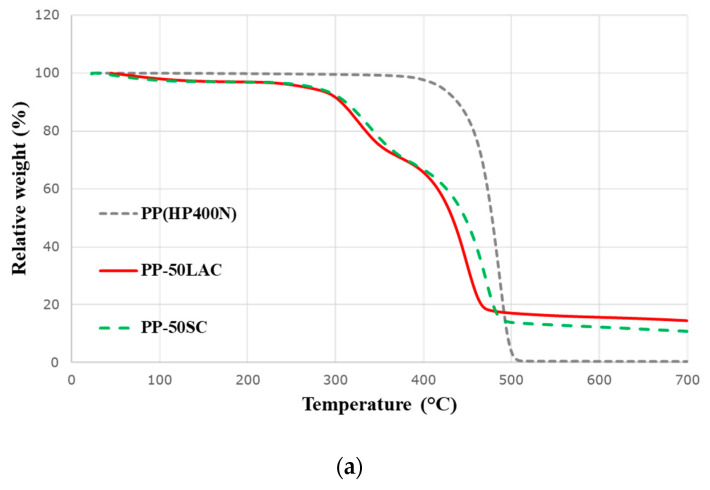

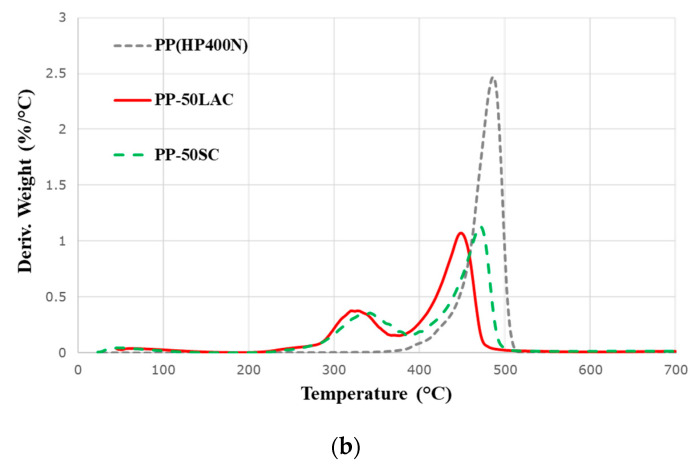
(**a**) Thermogravimetric and (**b**) derivative thermogravimetric curves of neat PP and PP–casein (LAC/SC) samples in nitrogen.

Thermal decomposition of PP and PP–casein composites is also demonstrated in Figure 4 and Table 3, respectively. The decomposition of the neat PP is a single phase with the maximum loss rate at 487.2 °C in Figure 4a and Table 3. However, both PP–LAC and PP–SC composites have two decomposition phases with the 14.5% and 10.8% final residue at 700 °C, respectively. According to Table 3, the first decomposition phase (T_max1_) of PP–LAC and PP–SC composites is driven by the decomposition of casein (LAC and SC) and the second decomposition phase (T_max2_) is related to the decomposition of PP. As shown in Figure 4b and Table 3, the PP–LAC composite has the maximum mass loss rate at 448.7 °C, whereas the highest decomposition rate of PP–SC composite is at 471.0 °C. It can be concluded that LAC decomposes at the slightly earlier stage and then dehydration and cross-linking reactions of LAC under the thermal degradation can more produce the char residue than SC in the PP-based composite. The onsets of the decomposition temperatures of PP–LAC and PP–SC composites are lower than that of PP because the casein’s decomposition occurs at an earlier stage compared to PP, Figure 3b and Table 3. Nevertheless, both casein products successfully decrease the pyrolysis rate of each composite from 2.5%/°C to about 1.1%/°C, which supports that casein products have the contributing effect on delaying the degradation of the PP composites.

**Figure 5:**
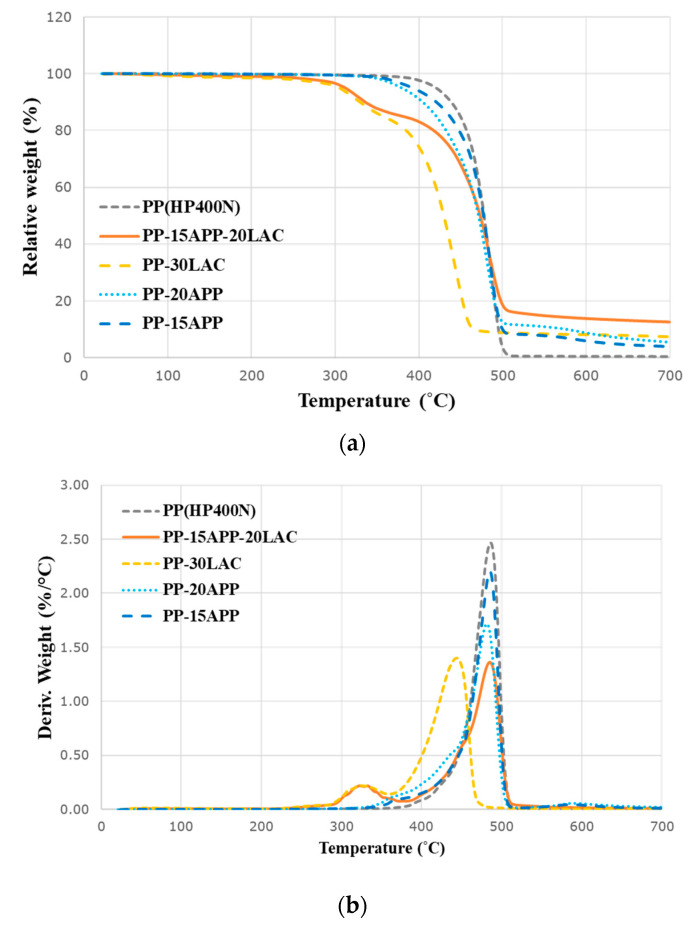
(**a**) Thermogravimetric and (**b**) derivative thermogravimetric curves of neat PP, PP–LAC, and PP–LAC–APP samples in nitrogen.

The combined effects of LAC and APP on the thermal decomposition of the composite have been demonstrated in Figure 5 and Table 3. The addition of LAC and APP clearly increases the amount of the final residue of the composite (12.6%) compared to one of the PP-20APP sample (5.5%). In comparison between PP-15APP-20LAC and PP-15APP, it is obviously noticed that 20% of LAC has improved the final residue amount from 3.9% to 12.6%. In terms of the two different decomposition phases in PP-15APP-20LAC, the first decomposition phase is related to T_max1_ of LAC, while the second decomposition phase is driven by the decomposition of APP based on the data in Table 3. The difference in T_max2_ between those of PP-30LAC and PP-15APP-20LAC can validate that the second decomposition phase in PP-15APP-20LAC is driven by the decomposition of APP. The onset of the decomposition of PP-15APP-20LAC occurs at a lower temperature than those of neat PP and PP–APP composites due to the incorporation of LAC (20 wt%). According to Figure 5b, it can be clearly seen that the pyrolysis rate of PP composites including LAC are lower than both of PP–APP samples. Specifically, the maximum rate of PP-20APP is around 1.7 %/°C, while one of PP-15APP-20LAC is around 1.4 %/°C. It can be highlighted that LAC can encourage the reduction of the maximum rate of the composite’s pyrolysis.

### 3.3. Cone Calorimeter Results

Cone calorimeter tests have been conducted to understand the ignitibility and the combustion behaviour of the samples under heat radiation. Table 4 illustrates cone calorimeter data of the testing samples, such as TTI, PHRR, TPHRR, THR, and smoke production. 

**Figure 6:**
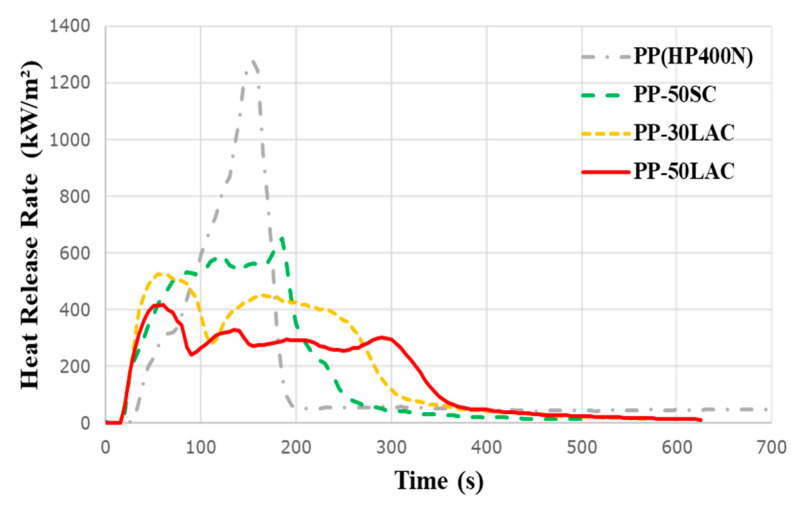
Heat release rate (HRR) curves of neat PP, PP-50% LAC/SC, and PP-30% LAC samples.

Heat release rate (HRR) is one of the important parameters to investigate the flame characteristic of a material because it contributes to the rate of fire growth, the size of the fire, and an effective heat of combustion [24]. As demonstrated in Figure 6, the neat PP demonstrates the highest PHRR (~1250 kW/m^2^) with a narrow peak due to the intensive combustion during the testing. In contrast, the incorporation of casein results in the significant reduction of the PHRR. In particular, the PP-50SC shows around 48% reduction of PHRR (~650 kW/m^2^), while the addition of 50% LAC achieves around 66% reduction of PHRR (~420 kW/m^2^) compared to a neat PP. Moreover, the time of PHRR between PP-50SC and PP-50LAC are different with about 180 and 60 s, respectively. The PHRR of PP-50SC at the end of the test can be explained that a continuous heat radiation from a cone heater destructs the char surface; hence, the HRR continually increases until the peak point. In previous research, it has been reported that the increasing amount of sodium as part of FR agents does not encourage the reduction of PHRR of the composite [25]. For PP-50SC, the presence of sodium could adversely affect the char-forming reaction in respect with Lewis acid approach to catalyse the chelation with the Lewis base that leads to the cross-linking reaction [26]. On the other hand, the HRR curve of PP-50LAC can indicate that the composite reaches the peak point during the char formation and gradually reduces the HRR without any obvious PHRR due to the compact char surface. It can be considered that inorganic phosphate and isocyanic acid can discharge under the thermal decomposition to promote the dehydration and cross-linking of polymer end chains and reactive groups to produce carbonaceous residue [27]. Moreover, carboxyl/sialic acid groups in κ–casein molecules that are located on the surface of the casein can facilitate the charring process [28]. The HRR curve of the PP-50SC sample matches with a typical HRR curve of an intermediate thick non-charring material, whereas the PP-50LAC sample’s result corresponds to the HRR curve of a thermally thick charring material [29].

The addition of 30 wt% of LAC also leads to a 58% decrease in PHRR compared to that of PP and lower HRR than PP-50SC. As a result, it can be concluded that LAC obviously has a better FR performance than SC in respect of the reduction of the composite’s PHRR. In addition, the comparison of the PHRR of PP-30LAC (~530 kW/m^2^) with other protein fibre composites (e.g., wool–PP composite: ~770 kW/m^2^ and chicken feather–PP composite: ~1230 kW/m^2^) reveals that LAC possesses a better natural fire resistance [30,31].

**Figure 7:**
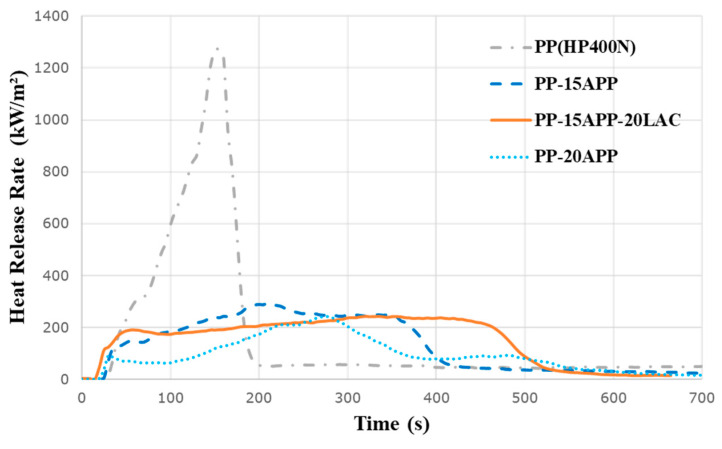
HRR curves of neat PP, PP-15%/20% APP, and PP-15%APP-20%LAC samples.

The effect of the combination of LAC and APP on the HRR of PP is evaluated in Figure 7. The PP-15APP-20LAC sample demonstrates a notable reduction (~80%) of PHRR compared to that of neat PP. Moreover, the HRR curve of PP-15APP-20LAC is more stable without any obvious peaks than other samples. In contrast, PP samples including only APP show the sharp peaks of HRRs at the middle of the curves as the char was not stable under the heat flux. More specifically, the loading of 20 wt% of LAC into PP-15APP composite results in the further reduction of the PHRR (~248 kW/m^2^) in comparison with one of PP-15APP. Furthermore, a comparison with other synthetic FR systems puts more credit on the influences of LAC and APP on the HRR reduction. For example, Wang et al. [32] used 15% synthesised FR compounds with 15% APP, resulting in around 63% reduction of the PHRR compared to that of neat PP. Besides, the PHRR value of PP-15APP-20LAC is better compared to that of PP-based composites including wool and CF fibres treated with IFR constituents, namely ethylenediamine phosphate (EDAP) and phosphoric acid (PA). In particular, the PHRRs of PP composites including 40% of wool and CF fibres that contain 14% EDAP and 26% wool and CF fibres with 6% PA were ~430 kW/m^2^ and ~330 kW/m^2^, respectively [9].

Two major gaseous species, carbon monoxide (CO) and carbon dioxide (CO_2_), were measured during combustion. As shown in Table 4, PP–LAC composites show the increasing yield of CO and the decreasing yield of CO_2_. At the same time, it can be seen that the PHRR value of PP–LAC composites decreases. By contrast, the yield of CO of PP-50SC does not increase even though the yield of CO_2_ and the value of PHRR decrease compared to one of a neat PP. It has been known that the incomplete combustion due to the char formation can induce the increase in the yields of CO and the decrease in the yield of CO_2_ and the value of the PHRR, respectively [33,34]. As a result, it can be mentioned that the charring ability of LAC is superior to one with SC to hinder the combustion cycle.

**Figure 8:**
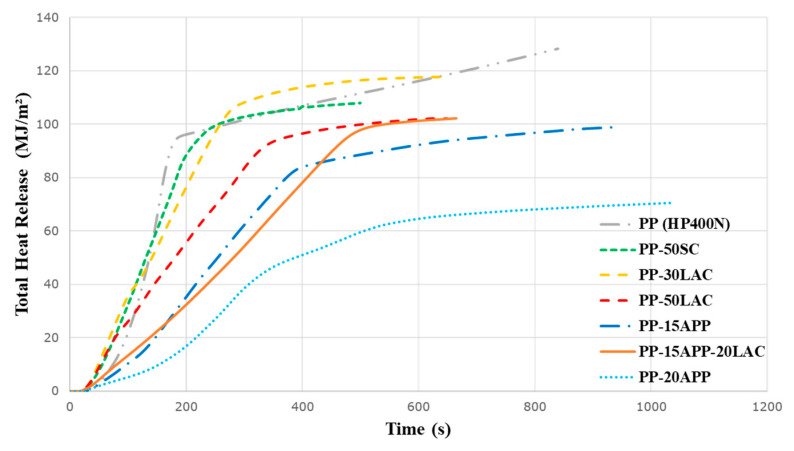
Total heat release (THR) curves of neat PP, PP-30%LAC, PP-50% LAC/SC, PP-15%/20% APP, and PP-15%APP-20%LAC samples.

Positive effects of the casein on total heat release (THR), which is the integral of the HRR curve with respect to time, of testing samples are demonstrated in Table 4 and Figure 8. The PP-50LAC composite produces less heat (~101 MJ/m^2^) than that of neat PP (~129 MJ/m^2^). In terms of the gradient of the THR curve, they can be interpreted as representative of flame spread [35,36]. Obviously, the gradient of PP-50LAC curve is lower compared to one of PP-50SC so that it can be assumed that LAC has a better ability to suppress the spreading flame during the combustion due to the stable and rigid char formation. This effect is also appeared in the comparison between PP-15APP and PP-15APP-20LAC composites. The addition of 20 wt% LAC into PP-15APP composite minimises the spreading flame of the composite compared to that of PP-based composite including only 15 wt% APP.

The standard deviation values of the cone calorimeter data are also shown in Table 4. All the parameters except for the TPHRR of PP-15APP sample have the coefficient of variation within 10%. The unstable char formation of the PP sample with low amount of APP could induce random occurrence of damages on the char surface, thereby varying the time to PHRR. However, the low standard deviation of overall data indicates that the testing results are highly accurate and reliable. 

### 3.4. Char Formation

**Figure 9:**
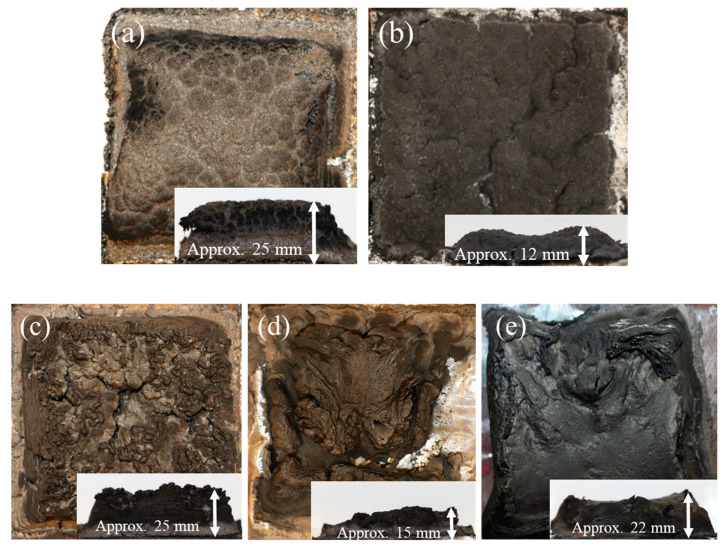
Top and side views of char residues: (**a**) PP-50LAC, (**b**) PP-50SC, (**c**) PP-15APP-20LAC, (**d**) PP-15APP, and (**e**) PP-20APP samples.

Figure 9a,b show char formation of the PP-50LAC and PP-50SC composites, respectively, whereas neat PP is completely burnt without any residues. The height of the char from PP-50LAC is remarkably higher by 13 mm than one of PP-50SC. Furthermore, the char-crack can be observed on the surface of PP-50SC, while the char of PP-50LAC has the compact surface without any damages. The charring and forming ability of the LAC contributed to the reduction of HRR, but still limited to form a rigid char structure. The combined effects of LAC and APP on the formation of rigid and voluminous char are identified in Figure 9c. The char of PP-15APP added 20% LAC composite does not show any severer damages from top and sides. On the other hand, the residue of PP-15APP composite collapses during the testing, Figure 9d. Furthermore, the char height of PP-15APP added 20% LAC composite is higher than one of PP-20APP by 3 mm. In addition, the deformation of char sides can be observed from the PP-20APP sample, Figure 9e. Therefore, it is evident that the incorporation of the casein conducts an important role in improving mechanical resistance of the composite’s char under heat flux.

### 3.5. Vertical Burn Test (UL-94)

The UL-94 vertical burn test is designed to evaluate the burning characteristics of various plastics under the flame expose by the visual observation. As demonstrated in Table 5, the neat PP resulted in the no rating (NR) as the drips of the specimen began in 2–3 s after the first flame impingement and the flame reached a holding clamp. The PP composites based on the different casein products also burnt substantially more than 30 s up to the clamp, but the composites dripped the residue in around 40 s after the first flame application. Furthermore, a cotton below the sample gets ignited due to the flaming drips. The burning behaviour can be related to the formation of carbonaceous char around the bottom and sides of the sample, but the weak char caused the flame penetration into the sample and continuous burning.

**Figure 10:**
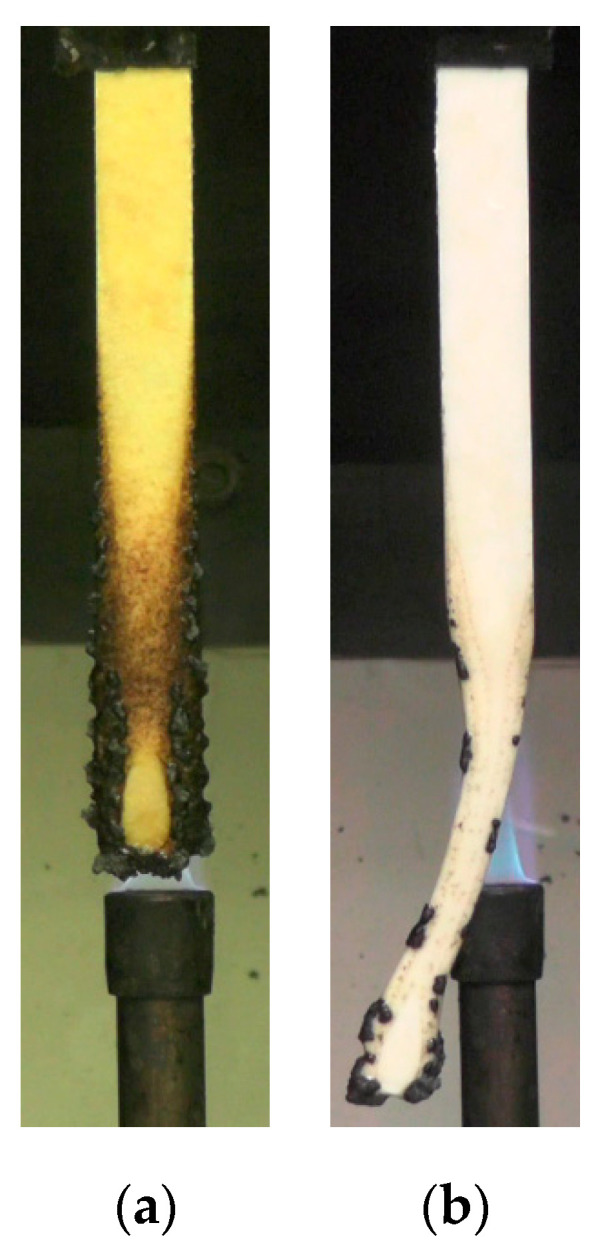
Samples after vertical burn tests: (**a**) PP-15% APP-20% LAC and (**b**) PP-20% APP composites.

In contrast, the combined effects of LAC and APP rendered a positive outcome. The PP-15APP-20LAC achieved the highest level of burning resistance with V-0 rating since the flame was extinguished without drips soon after the 10 s flame applications, Table 5. Figure 10a shows that the sample after the flame out still maintains its integrity without melting and deforming, whereas the PP-20APP sample is considerably distorted due to the afterglow, Figure 10b.

### 3.6. Tensile Properties

**Figure 11:**
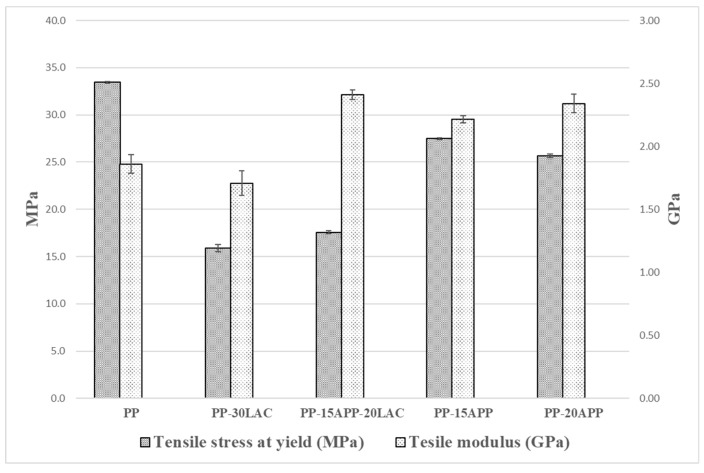
Tensile properties of neat PP, PP-30% LAC, PP-15% APP-20% LAC, and PP-15/20% APP samples.

Tensile strengths and moduli of a neat PP and PP composites are demonstrated in Figure 11. As PP composites, PP-30LAC, PP-15APP-20LAC, PP-15APP, and PP-20APP were chosen to understand the effect of LAC on tensile properties. The addition of 20 wt% of LAC with 15 wt% of APP improves the stiffness of the composite by 23% compared to a neat PP. Moreover, the improvement of the stiffness by the combination of LAC and APP was better than one of PP composites including APP only. On the other hand, the adverse effects of the casein on the tensile strength are identified. It can be considered that the particle size (310 µm) and loading of LAC affected the reduction of tensile strength values of the PP-30LAC and PP-15APP-20LAC composites [37,38]. Additionally, the hydrophilic LAC might cause a poor compatibility with the hydrophobic polymer [39]. The relative standard deviations of the tensile strength and modulus values from the samples are within 2% and 6%, respectively. The low values can imply the high reliability of the testing results.

### 3.7. Morphology

**Figure 12:**
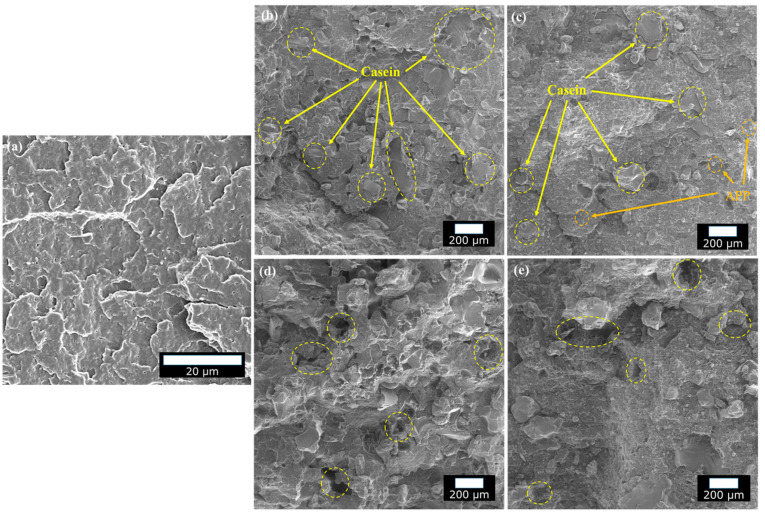
SEM images: cryofractures cross-sections of (**a**) neat PP, (**b**) PP-30LAC, and (**c**) PP-15APP-20LAC, and tensile fractured cross-sections of (**d**) PP-30LAC and (**e**) PP-15APP-20LAC.

The cryofractured cross-sections of PP, PP–LAC, and PP–APP–LAC composites are shown in Figure 12a–c, respectively. Reasonably well distributed LAC particles in the polymer can be identified in Figure 12b. Moreover, the PP-15APP-20LAC composite shows a good dispersion of the APP particles along with LAC, Figure 12c. It is to be noted that the dispersion states of the additives in the composites conducts an important role in improving the fire performance. On the other hand, the tensile fractured cross-section of the PP–LAC composite, Figure 12d, demonstrates several holes (yellow circles) due to the pull-out traces of LAC particles after the tensile test. In addition, the detrimental influence of the APP particles on the interfacial behaviour is detected in Figure 12e. Hence, the reduction of tensile strength values of the composites may be mostly attributed to the poor interfacial adhesion between the additives and PP.

## 4. Conclusions

In the present work, two casein products, e.g., LAC and SC, were employed to investigate their effects on the flame retardancy and mechanical performance of the PP composites, which were produced using melt-blending and compression moulding processes. DSC results demonstrate that casein products increase the melting and crystallization temperatures of the composites. The increase of the final char residue and the reduction of the pyrolysis rate of the PP–casein composites are clear from the TGA study. The cone calorimeter results of these composites also reveal that LAC has better flame-retardant performance than SC, showing a 66% lower value of PHRR (~420 kW/m^2^) and the generation of a more compact char. It is important to note that the addition of 30 wt% LAC can achieve a 58% reduction of PHRR compared to neat PP and better fire reaction properties compared to other protein-based PP composites. Moreover, the combined effects of LAC and APP can be clearly demonstrated by a further decrease in the PHRR value (~248 kW/m^2^) with self-extinguishment (V-0) under the vertical burn test. The considerable reduction of PHRR reveals that 20 wt% of LAC can play a significant role in forming the compact and rigid char of the composite. Besides, the combination of 20 wt% of LAC and 15 wt% of APP also improve the composite’s tensile modulus by 23%. However, LAC has an adverse influence on the tensile strength because of its large particle size and inferior particle/matrix interfacial bonding. Considering all the presented results, it can be said that the casein has a great potential to be an effective flame retardant derived from a natural source.

## Figures and Tables

**Table 1:** The composition of all polypropylene (PP) composite samples.

Samples	PP (%)	LAC (%)	SC (%)	APP (%)
PP(HP400N)	100	-	-	-
PP-50SC	50	-	50	-
PP-50LAC	50	50	-	-
PP-30LAC	70	30	-	-
PP-15APP	85	-	-	15
PP-20APP	80	-	-	20
PP-15APP-20LAC	65	20	-	15

**Table 2:** Differential scanning calorimetry (DSC) data information about neat PP, casein (LAC/SC), and PP composite samples.

Samples	T_g_ (°C)	T_m_ (°C)	T_c_ (°C)
PP(HP400N)	-	154.7	119.6
SC	197.58	-	-
LAC	193.94	-	-
PP-50SC	-	156.1	121.1
PP-50LAC	-	156.3	121.4
PP-30LAC	-	156.5	121.4
PP-15APP	-	158.1	126.8
PP-20APP	-	157.9	128.4
PP-15APP-20LAC	-	158.2	128.1

**Table 3:** Thermogravimetric analysis (TGA) data of neat PP, casein (LAC/SC), and all PP composite samples.

Samples	T_onset_	T_max1_	T_max2_	Residue at T_max1_ (wt%)	Residue at T_max2_ (wt%)	Residue at 700 °C (wt%)
PP(HP400N)	385.6	487.2	-	30.4	-	0
SC	277.4	337.2	-	63.8	-	22.3
LAC	279.1	328.6	667.2	64.7	20.0	15.3
PP-50SC	276.4	334.3	471.0	82.4	29.8	10.8
PP-50LAC	271.2	323.1	448.7	84.2	34.9	14.5
PP-30LAC	302.4	325.1	443.7	91.9	32.7	7.4
PP-15APP	388.9	485.9	-	33.4	-	3.9
PP-20APP	377.6	483.3	-	33.1	-	5.5
PP-15APP-20LAC	308.5	324.4	484.6	91.6	34.1	12.6

**Table 4:** Cone calorimeter data of neat PP, PP–casein (LAC/SC), and APP samples.

Samples	TTI (s)	PHRR (kW/m^2^)	TPHRR (s)	THR (MJ/m^2^)	CO (kg/kg) (± 0.002)	CO_2_ (kg/kg) (± 0.06)
PP(HP400N)	27.5 ± 1	1253.91 ± 27	150 ± 7	128.50 ± 3	0.029	2.52
PP-50SC	18.6 ± 2	652.49 ± 9	185 ± 0	105.26 ± 3	0.023	2.06
PP-50LAC	18.0 ± 1	421.70 ± 4	57 ± 6	101.23 ± 1	0.035	1.95
PP-30LAC	16.3 ± 1	529.34 ± 5	60 ± 5	118.27 ± 1	0.033	2.22
PP-15APP	22.3 ± 2	298.32 ± 6	258 ± 79	96.10 ± 8	0.032	2.08
PP-20APP	19.0 ± 0	243.14 ± 28	273 ± 4	70.25 ± 5	0.036	1.53
PP-15APP-20LAC	14.7 ± 1	248.00 ± 12	355 ± 35	101.75 ± 1	0.043	1.94

**Table 5:** UL-94 data of neat PP, PP–casein (LAC/SC), and APP samples.

Samples	T_1_^a^ (s)	T_2_^a^ (s)	T_t_^b^(s)	T_a_^c^ (s)	AA_h_^d^	CI_d_^e^	Classification
PP(HP400N)	112.2	-	-	-	Yes	Yes	NR
PP-50SC	67.2	-	-	-	Yes	Yes	NR
PP-50LAC	80.6	-	-	-	Yes	Yes	NR
PP-30LAC	66.2	-	-	-	Yes	Yes	NR
PP-15APP	4.6	66.0	70.6	-	Yes	Yes	NR
PP-20APP	0	0	0	-	No	No drips	V-0
PP-15APP-20LAC	0	0	0	-	No	No drips	V-0

^a^ Afterflame time of the first (**T_1_**) and second (**T_2_**) impingement of each sample; ^b^ Total afterflame time of each sample (**T_t_**); ^c^ Afterflame plus afterglow time of each sample (**T_a_**); ^d^ Afterflame or afterglow of any samples up to the holding clamp (**AA_h_**); ^e^ Cotton indicator ignited by flaming particles or drops (**CI_d_**).
